# Barriers to accessing maternal health care amongst pregnant adolescents in South Africa: a qualitative study

**DOI:** 10.1007/s00038-020-01374-7

**Published:** 2020-05-09

**Authors:** Michelle Olivia Erasmus, Lucia Knight, Jessica Dutton

**Affiliations:** School of Public Health, University of Western Cape, Cape Town, South Africa

**Keywords:** Adolescent pregnancy, Adolescent sexual and reproductive health, Antenatal care, Reproductive health in South Africa, Stigma, Gender

## Abstract

**Objectives:**

This study explores the barriers to accessing antenatal care (ANC) services amongst pregnant adolescents within a particular community of South Africa.

**Methods:**

An exploratory qualitative design was applied to examine the views of pregnant adolescents. In-depth interviews were conducted with pregnant adolescents at the Mitchells Plain Midwifery Obstetric Unit, as well as nursing staff working at the facility. Thematic analysis was then used and analysis was framed using the social–ecological model for health-seeking behaviour.

**Results:**

This study found that barriers to adolescents seeking ANC often centered on a discourse of adolescent pregnancy being deviant, irresponsible, and shameful. Pregnant adolescents often absorbed these beliefs and were fearful of other’s reaction within their family, the community, at school, and within the ANC facilities.

**Conclusions:**

Stigma regarding adolescent pregnancy participates in the perpetuation of a culture of non-disclosure and shame, which stands in the way of young pregnant people seeking the care they require. Such beliefs and attitudes need to be challenged at a community and national level.

**Electronic supplementary material:**

The online version of this article (10.1007/s00038-020-01374-7) contains supplementary material, which is available to authorised users.

## Introduction

South Africa’s (SA) adolescent birth rate is high, with 49 per 1000 births (The World Bank 2015). Maternal disorders are the second leading cause of death amongst adolescent girls, and adolescents face a higher risk of complications and death from pregnancy and childbirth than older women (AbouZahr 2013; WHO 2014). Early adolescent pregnancy is also associated with increased HIV incidence (Christofides et al. 2014). Adolescents have shown slower uptake of antenatal antiretroviral (ART) and higher mother-to-child transmission of HIV (Fatti et al. 2014). Adolescents face barriers to accessing maternal health services creating missed opportunities for HIV testing and treatment, and early detection of potential complications. Early access to antenatal care (ANC), amongst pregnant adolescents, is therefore a priority in reducing maternal morbidity and mortality (Tsawe and Susuman 2014; Atuyambe et al. 2008).

In South Africa (SA), adolescent pregnancy is most often depicted in negative terms, conjuring up the image of an impoverished teen who’s impeded bright future acts as a burden to community and national development (Ngabaza 2011; Mkhwanazi 2012; Ngabaza and Shefer 2013; Macleod and Feltham-King 2019). Discourse on adolescent pregnancy and parenting centers around notions of deviance, irresponsibility, and shame (Mkhwanazi 2012). This discursive framework, as well as the embedded gendered norms within it, is actively challenged by some civil society, scholarly work, and pregnant teens themselves (Ngabaza and Shefer 2013; Macleod and Feltham-King 2019). In challenging the dominant discourse on adolescent pregnancy, there is a move to engage with the very real and potentially harmful outcomes of being young and pregnant, while simultaneously not inflicting harm. This article situates itself within the local and global conversation that attempts to offer alternatives to the punitive and moralistic language often applied to adolescent pregnancy.

The sexual and reproductive health (SRH) rights of adolescents in SA are protected by law. National policy exists to prevent discriminatory practices that exclude pregnant students from education, and maternity care within government health facilities is available free of charge, yet adolescents still face numerous barriers to accessing SRH services (Macleod and Feltham-King 2019; Cooper et al. 2016; Hoopes et al. 2015; Ngabaza and Shefer 2013). Pregnant adolescents often encounter violence, anger, and breakdown in relationships with parents or caregivers as a result of their pregnancy (Hill et al. 2015; Phafoli et al. 2007; Ilika and Anthony 2004). The fear that accompanies disclosing one’s pregnancy to parents or guardians also deters early ANC uptake (Ngabaza 2011). Mistreatment by or negative attitudes of healthcare workers (HCWs) and schoolteachers have also been identified as barriers to access to healthcare amongst pregnant adolescents (Jewkes et al. 2009; Durojaye 2009; Ngabaza and Shefer 2013). These attitudes may be adopted to discourage sexual activity amongst adolescents and reflect deeply set patriarchal societal norms that punish particular reproductive and sexual behaviour based on cultural standards and socio-economic status (Amroussia et al. 2017; Godia et al. 2014; Alli et al. 2012). Delays in disclosure of pregnancy may lead to delays in adolescents seeking ANC, increasing the chances of pregnancy related complications going undetected (Chaibva et al. 2009).

This paper aims to explore the perceptions and experiences of pregnant adolescents utilizing one Midwifery Obstetric Unit (MOU) in urban Western Cape for their maternal health needs, in order to understand and explore barriers to access to care amongst pregnant adolescents within this specific context. MOUs are free midwifery run maternal health facilities comprised of an antenatal clinic, labour ward, and postpartum care for women and newborns. The social–ecological model (SEM) of health-seeking behaviour is used in this paper to frame the results. The theory suggests that factors at the individual level, community and structural level interact to shape health-seeking behaviour (Sallis et al. 2008).

## Methods

This study used an exploratory qualitative study design. Applying purposive sampling to select participants, semi-structured interviews were conducted with 10 adolescents who were English or Afrikaans speaking, pregnant, and between the ages of 16 and 19 years. The lead researcher designed and followed a semi-structured interview guide (see appendix 1), which was informed by examining the methodology and literature in the field as well as through consultation with the research supervisor. The interviews took place in a private space at the clinic by the lead researcher, who has undergone qualitative research training. Two nursing staff, with at least 2-year working experience at an MOU, were also interviewed as key informants.

Interviews were audio recorded and the lead researcher transcribed and analysed all interviews. The data was analysed manually using thematic analysis (Robson 2011). The analysis started with familiarisation with the data through transcription and multiple readings of the transcripts. The lead researcher then coded the transcripts and proceeded to organise and sort the codes into themes. Data analysis was an iterative process and included reflection to ensure rigour. In addition, data sources were triangulated through the inclusion of both pregnant adolescents and nursing staff. The study design, findings and interpretation of data were checked and discussed with the research supervisor for peer debriefing to minimise bias and ensure rigour. The University of the Western Cape Biomedical Research Ethics committee provided ethics clearance and permission for the research was granted by the Western Cape Department of Health. Data collection began with a thorough informed consent process. Before the interviews were conducted, consent was given by all participants, and for participants under 18, consent was also sought from a parent or guardian.

This research was conducted in Mitchells Plain in the Western Cape, South Africa. The community has high levels of crime, unemployment and poverty (Department of Provincial and Local Government South Africa 2011). It has a birth rate of 54.6 per 1000 births amongst girls under 18 years of age (Western Cape Government Provincial Treasury 2013).

## Results

Pregnant adolescents in this study identified numerous barriers and facilitators for access to maternal health care. The analysis and presentation of the results is framed by the social–ecological model (SEM) of health-seeking behaviour. The theory suggests that factors at the individual level, which may include intra and interpersonal, community and structural level interact and shape health-seeking behaviour (Sallis et al. 2008) including uptake of ANC (Table 1).

**Table 1:** The demographic profile of participants, South Africa, 2017

Age (*n* = 10)	
16	3
17	1
18	1
19	5
Relationship status (*n* = 10)	
Married	2
Engaged	1
In a relationship with boyfriend	4
Unmarried but relationship status not specified	3
Educational status (*n* = 10)	
Completed secondary schooling	2
Left school before completing school	5
Still attending school	3

### Individual factors

The individual level included emotional factors and knowledge regarding maternal health services. Although these factors acted at an individual level to influence the health-seeking behaviour, they were also heavily influenced by community and societal norms and expectations.

### Negative emotional response to pregnancy

Participants indicated that they experienced negative emotional responses such as sadness, fear and guilt when they realised that they were pregnant.

*[I felt] sad… and guilty [when I found about the pregnancy]…because I am still young.* age 16

These negative responses were most likely a reaction to deviating from internalised societal norms possibly resulting in young women delaying ANC attendance. Unwanted pregnancy or being in denial of the pregnancy may also have led to delays in attendance.

*Actually, I didn’t want to come [to the MOU], you see… my fiancé, he did tell me I must come so that we can find out, you see, before it’s too late. So I told him OK then… Because I was scared, I don’t want to know [about my pregnancy] actually*. age 19

### Lack of knowledge

Knowledge about the availability and recommended timing of access to ANC services also influenced adolescent uptake of these services.

*I knew nothing about it [ANC attendance], I didn’t even know about this clinic, so it was my first time coming here*. age 18

Many participants reported that they had little knowledge regarding general maternal health care.

*Yes, [I] actually [know] a little bit [about maternal health]. You must eat healthy food and you must drink a lot of water, and you mustn’t move…you mustn’t have heavy stuff on you when you pregnant you see, so not actually much about the baby*. age 19

They therefore did not realise the need for ANC attendance.

### Interpersonal factors

#### Fear of disclosing pregnancy

Family and social norms played a crucial role in access to ANC services. A common thread amongst the participants was fear of pregnancy disclosure to parents or guardians for fear of bringing shame to the family. Pregnant adolescents anticipated a negative response to their disclosure and therefore delayed it.

*… at first I was scared to tell [my parents]…about my pregnancy. Then I did not want to come book [at the MOU for initial visit] or anything. I wanted actually my mom to come with me, like now. Just…I was scared to tell them that is why I did not book before the time.* age 17

Many participants only attended the MOU after their parents or caregivers knew about the pregnancy:

“[I came to the clinic] *because my mom found out [about the pregnancy]…the last two months I didn’t tell, I didn’t tell her… She told me to come here, so that I will know sooner, if the baby has a problem or not …”* age 18

A delay in pregnancy disclosure thus may lead to a delay in accessing ANC services amongst pregnant adolescents.

#### Fear of punishment

Some participants expressed fear of punishment after pregnancy disclosure:

*Maybe [other pregnant girls] mothers’ abuse [them], others [say] ok, you are pregnant, and [still young, so] take all your clothes [and] go. Maybe you going to go where? Which place [are] you going to go? [The] only [place you have to live] is your home. Just imagine going outside, your clothes is not in the suitcase, it’s just in a plastic bag…*. age 16

These delays in disclosure led to a delay in attending ANC for some young women. Nurses at the MOU acknowledged that fear of pregnancy disclosure was a barrier to maternal health care amongst adolescents.

*Some of them [pregnant adolescents], they come to book [for delivery] late, just because they were hiding the pregnancy. But if you ask them then they will tell you that I did not want my mum to know, my mum didn’t know*. Nurse, age 57

Many participants reported that pregnancy disclosure was indeed met with anger from parents or guardians.

*I was scared to tell my mommy and daddy about [the pregnancy]…and when I told, my mommy saw that I got fat and that, so I told her [that I am pregnant] and then she got kwaad [angry], and afterwards she was happy. And my daddy didn’t talk to me… at all.* age 16

After disclosure, mothers seemed to change their mind to form a more positive impression, whereas fathers seemed to remain angry for longer:

*At first my mommy scold[ed] at me but then she overcame it, but…my daddy is still cross…a bit*. age 17

This shift in mindset by mothers and grandmothers served as a facilitator for access to maternal health services.

*My ma [grandmother] was disappointed [about the pregnancy] but…she said that…she…can’t do anything about it and that she will stand by me.* age 16

Mothers and grandmothers encouraged pregnant adolescents to attend the MOU.

*My mother told me that I should come [to the MOU] because…you need vitamins,* etc. age 19

This support and encouragement was an important facilitator such as below:

*[I knew I had to come to the MOU]…but I like, didn’t really take it seriously until my grandmother talked.* age 19

Pregnant adolescents in this study thus appeared to hold the advice of mothers and grandmothers to attend ANC in high regard.

### Community and social factors

#### Fear of stigmatisation and judgement

Participants in this study identified community and social factors as influencing adolescent’s access to ANC. Societal norms and expectations appeared to cut across to operate at both individual and interpersonal levels to determine health-seeking behaviour.

Many participants reported that fearing judgment from the community delayed attendance at ANC services.

*[I felt] nervous [about attending the MOU] because everybody is like looking at me, I am so young and stuff. [I feel] nervous, uncomfortable also… the young ladies sitting around me and…older people [are looking at me].* age 17

Participants were aware that premarital sexual activity would be met with disapproval from their community, and felt judged for defying religious and societal expectations. Participants therefore felt uncomfortable attending the MOU for their pregnancy, where older women and others in the community would be present.

Participants reported that attending the MOU before disclosing the pregnancy to their parents or guardians carried the risk of them being identified by community members, who may inform their parents or guardians of the pregnancy.

*I was, still afraid [to attend the MOU], someone walking past me that knows me [is going to] tell my parents [that I am pregnant], so… [I am] actually hiding a bit*. age 19

Adolescents expressed a connection between the stigma within the community and the fear of their parents being informed of their pregnancy. Fear of disclosing pregnancy at school was also a barrier to accessing health services. Pregnant adolescents were afraid to tell educators and peers about needing to miss school to attend the MOU, delaying their ANC attendance.

*Some of them [pregnant adolescents] they are still at school, they miss their appointment and then when you ask ‘Why didn’t you [come to the MOU]?’, [They say] “I was at school”, [When you ask pregnant adolescents] ‘But why didn’t you ask [for time off school to come to the MOU]?’ [They say] “I was scared”.* Nurse, age 57

Pregnant adolescents’ were in a position where they had to risk judgement when seeking ANC services.

*Already they [pregnant adolescents] have the expectation that somebody’s going to tell them what you are doing is wrong or somebody’s going to point a finger at them, somebody’s going to condemn them or judge them, you know.* Nurse, age 52

Fear of disclosing pregnancy due to community attitudes that adolescent sexual activity is immoral and shameful was thus a significant barrier for access to ANC services amongst pregnant adolescents.

### Organisational and health system factors

#### Negative perception of healthcare workers

At the system level, behaviour of HCWs influenced health-seeking behaviour. Most participants had a negative perception of HCWs at the MOU, and previous bad experiences.

*You know what, sometimes the younger we are ne [hey]? Sometimes we scared to come to the clinic, you know, because nurses, doctors, they shout at us, [they ask us] why you having pregnancy as young you are? Why? So we are scared [to come to the MOU].* age 16

Many participants had heard rumours about nurses being rude towards pregnant adolescents and therefore expected mistreatment from nurses resulting in delaying ANC access.

*I was scared [to come to the MOU] because, um, people thought, think that the nurses are very rude, but when I came here they were nice and they assisted me.* age 18

Nurses at the MOU were aware of the negative perceptions and identified this as a barrier for pregnant adolescent accessing care.

*You know everybody has got that attitude that the sisters are shouting at people, so…otherwise, if you explain everything to them [pregnant adolescents], everything just goes right.* Nurse, age 57

The negative perceptions and bad experiences of adolescents regarding HCWs made pregnant adolescents fearful of accessing maternal healthcare services.

## Discussion

The purpose of this study is to explore the barriers to accessing ANC services amongst pregnant adolescents. The findings showed that multiple factors interact to influence access to ANC services amongst pregnant adolescents. These include fear, stigma, and the negative perceptions of HCWs.

The SEM model for health-seeking behaviour highlights the interaction between individual, interpersonal, community and health system level factors interact to shape health-seeking behaviour (Sallis et al. 2008). The model failed to highlight the role of gender and gendered expectations as a barrier for seeking ANC amongst pregnant adolescents. Possibly because gender is so deeply ingrained in the above barriers and therefore hidden without the application of an intentional gender lens. This discussion therefore seeks to further elucidate the findings but with the application of a gender lens.

Individual factors delaying ANC attendance centred on the guilt and regret for being pregnant, the fear of repercussions from family and the wider community and a lack of knowledge of ANC. In many communities, cultural and religious rules dictate that pregnancy should only occur after marriage (Amroussia et al. 2017; Richter et al. 2006). While South Africa has high rates of fertility outside of marriage women may still face judgment and stigma when engaging in sexual activity at young ages and before marriage (Morwe et al. 2014). Unmarried pregnant adolescents often delay disclosing the pregnancy to conceal their deviation from cultural and religious expectations. These results suggest that younger adolescents, particularly those under the age of 17 years, seem to experience greater feelings of fear and shame than older adolescents. Internalizing the dominant discourse that deems pregnancy in young women as shameful speaks to the power of social norms to create a self-regulating population.

Before disclosing their pregnancy, the adolescents in this study felt guilty and amoral. This is similar to other research findings, with adolescents undergoing inflicting blame and shame on themselves before disclosing their pregnancy (De Villiers and Kekesi 2004). Feelings of fear also shaped adolescents attendance at an MOU. They expressed discomfort attending the MOU surrounded by older pregnant women, for fear of their judgement.

Feelings of shame and guilt are also housed within a cultural silencing of adolescent sexuality, leading to a lack of knowledge and information on what ANC involves and when to seek it (Ngabaza 2011). Misconceptions around adolescent sexuality, such as the belief that talking to young people about sexuality encourages them to become sexually active, creates an environment that stifles open dialogue. The idea that if it is not spoken about, then it is not happening carries dangerous consequences for adolescents and is premised on a system that privileges fundamentalism over freedom (Pino 2019).

On an interpersonal level, pregnant adolescents not only feared judgement from parents, but also punishment for becoming pregnant, such as expulsion from home. This may have been greater for younger adolescents who are more financially and otherwise reliant on family than those who are older. The interpersonal factors at the family level were linked to community influence through social and gendered norms regarding adolescent sexuality. Thus, deviation from societal norms regarding timing of pregnancy reflects negatively on the morals of the family (Richter et al. 2006; Wiemann et al. 2005). Fear of punishment from family cannot be attributed to internalised social and community norms alone, studies in the same context have revealed that pregnant adolescents have been expelled from their home as a form of punishment (Van Zyl et al. 2015). There are very real fears of being expelled from home, therefore, pregnant adolescents delay telling their parents, which in effect delays the process of attending ANC.

In SA, parental permission is not required to attend ANC. However, many adolescents fear that MOU attendance will lead to them being identified and their pregnancy being revealed to their parents, and therefore avoid attending ANC services prior to pregnancy disclosure. In saying this, participants in this study reported that once they had disclosed their pregnancy to their family, an initial negative response was often followed by support and advice from parents and guardians. Pregnant adolescents cited their mothers as pivotal in providing support and guidance through their pregnancy experience, including the necessity of attending ANC.

At an organisational level many adolescents expected scolding by nurses for being pregnant. This was a notable despite limited prior experience with maternal health care services and therefore resulting from negative hearsay (Loxton et al. 2007). This concern is justifiable, rooted in real events and experiences of pregnant women who attend MOUs. The frequency of such treatment is unknown, but there is evidence that HCWs demonstrate negative attitudes towards pregnant adolescents based on both age and marital status (Jonas et al. 2017) and it has been shown to be a barrier to accessing maternal health care amongst adolescents (Silal et al. 2012).

There are many factors that interact to influence health-seeking behaviour of adolescents. Anticipation of a negative response from family and the community, pregnant adolescents may delay pregnancy disclosure, placing them at risk of pregnancy complications that could be detected and attended to with ANC. The adolescents in this study only started attending ANC after disclosing the pregnancy to parents or caregivers although once they did their mothers and grandmothers played a pivotal role in encouraging their attendance at ANC (Mumah et al. 2014; Singh and Hamid 2015; Van Zyl et al. 2015). Fear of punitive measures and the association of adolescent pregnancy with shame intersect at all SEM model levels. The findings of this study, alongside similar studies, demonstrate the need to open up dialogue on adolescent sexuality. It has been suggested that a reparative reproductive justice model be applied in developing new and more compassionate ways of engaging with adolescent SRH (Macleod 2017, Macleod and Feltham-King 2019). According to Macleod and Feltham-King (2019), reproductive justice can be defined as an,

intersectional approach that locates analysis of health and reproduction within context. It forefronts, firstly, the intertwining of individual and social process, and, secondly, the complex interaction of power relations that cohere around various axes of discrimination… The reparative justice aspect of the framework highlights the need for social repair and support in the case of pregnancies where support is lacking and inequalities or injustices occur (Macleod and Feltham-King 2019, p. 2).

This framework gets to the heart of what participants shared in this study; the barriers they face are intertwined with the ways in which adolescent pregnancy is generally understood as always already negative. The discrimination that pregnant adolescents face due to gender inequality is also taken into consideration using this model through understanding the complicated ways power relations interact with SRH. Understanding the role of patriarchy within social expectations of girls and women helps unpack the ways in which punishing adolescent pregnancy or pregnancy outside of marriage acts as a form of control over the lives and bodies of women. The focus on social repair moves away from shaming women and girls for getting pregnant and towards societal changes that support adolescents and their sexual and reproductive needs.

### Implications for policy and programming

Age-appropriate, accessible, and innovative tools are required to assist pregnant adolescents with ANC attendance and self-care. The popularity of social media creates a platform where information regarding pregnancy, SRH and ANC can be made available while protecting one’s privacy. Change to community beliefs and behaviour requires time, where social media is rapid and fairly accessible. Commonly used cell phone apps, such as WhatsApp can be used to relay information regarding SRH. Information can be communicated through apps, empowering adolescents to make informed decisions regarding their SRH. Apps can be used to access counselling and locate safe spaces within their community. This in itself is not enough to break down the barriers created by stigma and judgement but such technology could offer adolescents a tool to better negotiate their SRH with regard to societal norms in a format they can access and are familiar with.

Communities, political leaders, and institutions need to address the ways in which they create and support harmful attitudes towards adolescent SRH. Images of adolescents practising agency and control over their own SRH need to become commonplace. When pregnant adolescents do not receive safe and adequate ANC their well-being is at risk. This study found that a major barrier to accessing ANC is facing the hurdles that shame, blame and stigma have built within the individual and community. Dismantling these barriers can reduce the rate of maternal mortality and morbidity, detect HIV and initiate ART when necessary, strengthen the prevention of mother to child transmission of HIV, improve the experience of pregnancy, and foster a culture of agency around adolescent SRH—all issues relevant at a local and global level.

### Limitations

This study included a number of possible limitations: the exclusion of adolescents who have not accessed a MOU, those below the age of 18 whose parents did not give permission, and those under 16 years were excluded from this study. A further limitation of this study is the inclusion of participants who speak English and Afrikaans only.

### Conclusion

Adolescent access to maternal health care is a complex issue. Gendered societal norms and stigma regarding adolescent pregnancy creates a culture of non-disclosure and shame around adolescent pregnancy, making it difficult for pregnant adolescent to access ANC. It is thus of utmost importance that a supportive environment be created for pregnant adolescents so that they can disclose pregnancy and access health care early. Such support needs to be extended to the entire family of a pregnant adolescent, shame towards the family acts to harm everyone. Efforts should be focused on educating communities and empowering pregnant adolescents to prevent unplanned pregnancies, increased knowledge on contraception and normalizing communication about sex, rather than shaming and disadvantaging adolescents for becoming pregnant. Comprehensive sexuality education needs to be implemented in all schools across the country to ensure learners have access to evidence-based information pertaining to their SRH.

## Electronic supplementary material

Below is the link to the electronic supplementary material.

Supplementary material 1 (DOCX 14 kb)

## References

[CR1] AbouZahr C (2013) Trends and projections in mortality and morbidity. In: WHO international conference on population development, women’s meeting. https://www.unfpa.org/sites/default/files/resource-pdf/Overview.pdf. Accessed 11 July 2015

[CR2] Alli F, Maharaj P, Vawda MY (2012). Interpersonal relations between health care workers and young clients: barriers to accessing sexual and reproductive health care. J Community Health.

[CR3] Amroussia N, Hernandez A, Vives-Cases C, Goicolea I (2017). “Is the doctor God to punish me?!” An intersectional examination of disrespectful and abusive care during childbirth against single mothers in Tunisia. Reprod Health.

[CR4] Atuyambe L, Mirembe F, Tumwesigye NM, Annika J, Kirumira EK, Faxelid E (2008). Adolescent and adult first time mothers’ health seeking practices during pregnancy and early motherhood in Wakiso district, central Uganda. Reprod Health.

[CR5] Chaibva CN, Roos JH, Ehlers VJ (2009). Adolescents mothers’ non-utilisation of antenatal care services in Bulawayo, Zimbabwe. Curationis.

[CR6] Christofides N, Jewkes R, Dunkle K, Nduna M, Shai N, Sterk C (2014). Early adolescent pregnancy increases risk of incident HIV infection in the Eastern Cape, South Africa: a longitudinal study. J Int AIDS Soc.

[CR7] Cooper D, Harries J, Moodley J, Constant D, Hodes R, Mathews C, Morroni C, Hoffman M (2016). Coming of age? Women’s sexual and reproductive health after twentyone years of democracy in South Africa. Reprod Health Matters.

[CR8] De Villiers FPR, Kekesi J (2004). Social interaction of teenage mothers during and after their pregnancy. S Afr Fam Pract.

[CR9] Department of Provincial and Local Government South Africa (2011) Mitchell’s plain nodal economic development profile. https://mitchellsplain.files.wordpress.com/2011/07/mitchells_20plain_20narrative.pdf. Accessed 15 July 2016

[CR10] Durojaye E (2009). Realizing access to sexual health information and services for adolescents through the protocol to the African Charter on the rights of women. Wash Lee J Civ Rights Soc Just.

[CR11] Fatti G, Shaikh N, Eley B, Jackson D, Grimwood A (2014). Adolescent and young pregnant women at increased risk of mother-to-child transmission of HIV and poorer maternal and infant health outcomes: a cohort study at public facilities in the Nelson Mandela Bay Metropolitan district, Eastern Cape, South Africa. S Afr Med J.

[CR12] Godia PM, Olenja JM, Hofman JJ, van den Broek N (2014). Young people’s perception of sexual and reproductive health services in Kenya. BMC Health Serv Res.

[CR14] Hill L, Maman S, Groves A, Moodley D (2015). Social support among HIV-positive and HIV-negative adolescents in Umlazi, South Africa: changes in family and partner relationship during pregnancy and the postpartum period. BMC Pregnancy Childbirth.

[CR16] Hoopes AJ, Chandra-Mouli V, Steyn P, Shilubane T, Pleaner M (2015). An analysis of adolescent content in South Africa’s contraception policy using a human rights framework. J Adolesc Health.

[CR17] Ilika A, Anthony I (2004). Unintended pregnancy among unmarried adolescents and young women in Anambra State, South East Nigeria. Afr J Reprod Health.

[CR18] Jewkes R, Morell R, Christofides N (2009). Empowering teenagers to prevent pregnancy: lessons from South Afirca. Cult Health Sex.

[CR19] Jonas K, Crutzen R, van den Borne B, Reddy P (2017). Healthcare workers’ behaviors and personal determinants associated with providing adequate sexual and reproductive healthcare services in sub-Saharan Africa: a systematic review. BioMed Cent Pregnancy Childbirth.

[CR20] Loxton D, Stewart Williams J, Adamson L (2007). Barriers to service delivery for young pregnant women and mothers: National Youth Affairs Research Scheme.

[CR21] Macleod C, Cherry A, Baltag V, Dillon M (2017). ‘Adolescent’ sexual and reproductive health: controversies, rights, and justice. International handbook on adolescent health and development.

[CR22] Macleod C, Feltham-King T (2019). Young pregnant women and public health: introducing a critical reparative justice/care approach using South African case studies. Crit Public Health.

[CR24] Mkhwanazi N (2012). A tough love approach indeed: demonising early childbearing in the Zuma era. Agenda Empower Women Gender Equity.

[CR26] Morwe KG, Klu EK, Tugli AK (2014). Teenage pregnancy in South Africa: a challenge to democracy. J Soc Sci.

[CR27] Mumah J, Kabiru CW, Izugbara C, Mukiira C (2014). Coping with unintended pregnancies: narratives from adolescents in Nairobi’s slums.

[CR28] Ngabaza S (2011). Teenage fertility and desire. Agenda Empower Women Gender Equity.

[CR29] Ngabaza S, Shefer T (2013). Policy commitments vs. lived realities of young pregnant women and mothers in school, Western Cape, South Africa. Reprod Health Matters.

[CR31] Phafoli SH, Van Aswegen EJ, Alberts UU (2007). Variables influencing delay in antenatal clinic attendance among adolescents in Lesotho. S Afr Fam Pract.

[CR32] Pino A (2019) Comprehensive sexuality education: why it matters. Daily Maverick, 1 Nov 2019

[CR34] Richter LM, Norris SA, Ginsburg C (2006). The silent truth of teenage pregnancies—birth to twenty cohort’s next generation. S Afr Med J.

[CR35] Robson C (2011). The analysis and interpretation of qualitative data.

[CR36] Sallis JF, Owen N, Fisher EB, Glanz K, Rimer BK, Viswanath K (2008). Ecological models of health behaviour. Health behaviour and health education: theory, research and practice.

[CR38] Silal SP, Penn-Kekana L, Harris B, Birch S, McIntyre D (2012). Exploring inequalities in access to and use of maternal health services in South Africa. BMC Health Serv Res.

[CR39] Singh S, Hamid A (2015). Reflections of a group of South African teenage mothers: sexual health implications. Health Educ J.

[CR40] The World Bank (2015) Data: adolescent fertility. www.data.worldbank.org/indicator/SP.ADO.TFRT/countries. Accessed 11 July 2015

[CR41] Tsawe M, Susuman A (2014). Determinants of access to and use of maternal health care services in the Eastern Cape, South Africa: a quantitative and qualitative investigation. BMC Res Notes.

[CR42] Van Zyl L, van der Merwe M, Chigeza S (2015). Adolescents’ lived experiences of their pregnancy and parenting in a semi-rural community in the Western Cape. Soc Work.

[CR43] Western Cape Government Provincial Treasury (2013) Regional development profile city of Cape Town. www.westerncape.gov.za/assets/departments/treasury/Documents/Socio-economic-profiles/dcO_city_of_cape_town_ke_6-dec_2013.pdf. Accessed 19 July 2015

[CR44] WHO (2014) Factsheet no 364. www.who.int/mediacentre/factsheet/fs364/en/. Accessed 11 July 2015

[CR45] Wiemann CM, Rickert VI, Berenson AB, Volk RJ (2005). Are pregnant adolescents stigmatized by pregnancy?. J Adolesc Health.

