# Supplementary material for: Barriers to accessing maternal health care amongst pregnant adolescents in South Africa: a qualitative study
Source: Int J Public Health. 2020 May 9;65(4):469–76. doi: 10.1007/s00038-020-01374-7 (PMC7275000; doi:10.1007/s00038-020-01374-7)
Supplement: Supplementary file 1 — Supplementary material 1 (DOCX 14 kb) [file 38_2020_1374_MOESM1_ESM.docx]

International Journal of Public Health

Article: Barriers to accessing maternal health care amongst pregnant adolescents in South Africa: a qualitative study

Electronic supplementary material

Appendix 1

**Interview guide- Adolescent**

• Introduce topic

The purpose of this research is to explore knowledge, attitudes beliefs and perceptions of pregnant adolescents regarding access to maternal health care services. The reason for this is that adolescents/young people often come to the MOU very late in pregnancy, so I want to find out reasons for this, and what makes it easier or more difficult for young people to come to the MOU. I hope that this research will then help to identify ways to improve services for young people.

• Gain demographic information (age, area of residence, school/employment, family background)

How old are you?

Where do you live?

Do you attend school/work/

If yes, what grade? If no, what grade did you finish?

Prompt for those who left school… Did you leave school due to pregnancy?

Who do you currently live with?

• Can you tell me about your experience of finding out that you were pregnant?

Probe: How did that make you feel? Can you tell me about whether anyone helped you with this and how?

• Can you tell me about why you came to the MOU?

Probe: Can you tell me about whether anyone helped you decide to come here?

Probe: Can you tell me about anything that happened that made you decide to come here?

• Can you tell me about what it was like coming here to the MOU for the first time?

Probe: How did you feel about coming to the MOU?

Can you tell me about any good experiences you had at the MOU? How did that make you feel?

Can you tell me about any bad experiences you had at the MOU? How did that make you feel?

• Can you explain if there is anything that made it easier for you to come to the MOU?

• Can you explain if there is anything that made it difficult for you to come to the MOU?

APPENDIX 2

**Interview Guide: Nursing staff**

• (Get demographic information: Age, years of experience)

• How do you feel about the high rate of teenage pregnancy in this community?

Probe: Can you tell me about things you think contribute to high rates of teenage pregnancy?

• Can you tell about why access to the MOU is important for pregnant teenagers?

• How do you feel about working with pregnant adolescents?

Probe: Can you tell me about any positive experiences/incidents when working with pregnant adolescents? How did that make you feel?

Can you tell me about any negative experiences/incidents when working with pregnant adolescents? How did that make you feel?

• How would you describe your interaction with pregnant adolescents at the facility?

Probe: Can you explain how you react to pregnant teenagers who miss appointments/don’t comply with medical advice?

Probe: Can you explain how you react to pregnant teenagers who seem nervous/anxious when they come to the MOU?

• Can you tell me about reasons why adolescents make late bookings?

Probe: Can you explain if there is anything at the MOU that contributes to teenagers making late bookings?

• Can you explain what you think would help adolescents’ access maternal health care?

Probe: Can you tell me about what you think staff can do differently to assist pregnant teenagers at the MOU?

Probe: Can you tell me about anything at the MOU you would like to see change to help you work more effectively with pregnant teenagers?
